# Application of Butylamine as a Conjugative Reagent to On-Column Derivatization for the Determination of Antioxidant Amino Acids in Brain Tissue, Plasma, and Urine Samples

**DOI:** 10.3390/ijms20133340

**Published:** 2019-07-07

**Authors:** Kamila Borowczyk, Patrycja Olejarz, Adrianna Kamińska, Rafał Głowacki, Grażyna Chwatko

**Affiliations:** Department of Environmental Chemistry, Faculty of Chemistry, University of Łódź, 163 Pomorska Street, 90-236 Łódź, Poland

**Keywords:** antioxidants, on-column derivatization, high performance liquid chromatography, biological samples

## Abstract

(1) Antioxidants are involved in body protection mechanisms against reactive oxygen species. Amino acids such as glutathione (GSH) and *N*-acetylcysteine (NAC) are known to be involved in providing protection against oxidative lethality. A quick and simple method for the determination of NAC and GSH in various biological matrices such as urine, plasma, and homogenates of brain tissues has been developed and described in this work. (2) The assay is based on reversed phase high performance liquid chromatography with spectrofluorimetric detection and on-column derivatization. Butylamine and *o*-phthaldialdehyde have been used as derivatization reagents. Since *o*-phthaldialdehyde constitutes a part of the mobile phase, the derivatization reaction and chromatographic separation occur simultaneously. (3) Linearity in the detector response for NAC in human urine was observed in the range of 5–200 nmol mL^−1^, and NAC and GSH in the brain tissue homogenates were observed in the range of 0.5–5 nmol mL^−1^ and 0.5–15 nmol mL^−1^, respectively. Human plasma linearity ranges covered 0.25–5.00 nmol mL^−1^ and 0.5–15 nmol mL^−1^ for NAC and GSH, respectively. The LODs for NAC and GSH were 0.01 and 0.02 nmol mL^−1^ while the LOQs were 0.02 and 0.05 nmol mL^−1^, respectively. The usefulness of the proposed method was proven through its application to real samples.

## 1. Introduction

Thiols belong to a group of organic compounds containing very active and biologically important sulfhydryl group (-SH). From a metabolic point of view, the most important thiols present in the human body are glutathione (GSH), cysteine (CYS), and homocysteine (HCY). Additionally, *N*-acetylcysteine (NAC) plays an essential role in the human body due to its metabolic and medical properties. All of these compounds are widespread in tissues and biological fluids such as plasma and urine [1,2]. These compounds play a crucial role in cell biology and pharmacology as well as the biochemistry of mammals. In living organisms, thiols are a substantial cellular component of many key pathways of metabolic processes. They play a significant role as active components of the sulfur cycle as well as take part in the defense mechanisms of the organism against the pathological effects of reactive forms of oxygen and nitrogen [1,3]. It has been proven that changes in some thiol concentrations and their molar ratio can act as potential indicators of cell health and the risk of diseases [2].

Antioxidants are known to be involved in body protection mechanisms against reactive oxygen species (ROS) produced by the reduction of oxygen to unstable free radicals like superoxide and non-radical species like hydrogen peroxide. Both enzymatic antioxidants (superoxide dismutase, catalase, and glutathione peroxidase) and non-enzymatic antioxidants (GSH, CYS, NAC, and α-lipoic acid) are involved in providing protection against oxidative lethality [4].

NAC is an acetylated precursor of the amino acid L-CYS and is endogenously present in biological systems. NAC is commercially available in many pharmaceutical formulations in intravenous, oral, or inhalation forms. It has been used in many clinical applications for over 50 years for the treatment of mucolytic disorders, inflammatory conditions of the respiratory tract, and in cases of paracetamol poisoning for the inactivation of the paracetamol’s toxic metabolites [5,6]. NAC is also used as a metal chelating agent for several toxic metals such as boron, chromium, cobalt, cadmium, arsenic, gold, and lead [5]. Evidence from in vitro and in vivo studies have indicated that NAC, by decreasing the extracellular concentration of the disulfide of CYS or its reduction to CYS as well as by being a donor of the sulfhydryl group, stimulates the synthesis of GSH, a ubiquitous source of the thiol group in the body [5,7].

GSH is the main intracellular tripeptide found in biological tissues. GSH participates in the transport of amino acids by the “glutamyl-cycle”, the detoxification of drugs, and also plays an essential role in protecting cells against the damaging effects of free radicals [3,8]. GSH is involved in regenerating other antioxidants (e.g., ascorbate) and is crucial for the detoxification of xenobiotics through its involvement in the mercapturic acid pathway [9]. GSH often attains millimolar levels inside cells, which makes it one of the most highly concentrated intracellular antioxidants [10].

If compared to other tissues, the brains of mammals consume the largest amount of oxygen, which means that ROS are still produced. GSH plays a key role in maintaining redox homeostasis in glial and neuronal cells and exerting a protective effect on cells, thus minimizing the oxidative effects of ROS. Unfortunately, in protective processes, GSH is depleted and for this reason, it is necessary to adjust its level. Among the three amino acids necessary for GSH synthesis (glutamate, glycine, CYS), CYS exists in the lowest concentration and its availability limits the rate of GSH production. Oral administration of CYS has little effect on GSH levels in the brain. Importantly, NAC leads to a rise in the level of CYS in plasma, resulting in an increasing plasma GSH concentration. There is evidence that NAC penetrates the blood–brain barrier by increasing GSH content in the brain cells [11,12,13].

Oxidative stress plays a key role in the pathogenesis of many diseases including cancer, inflammation, Alzheimer’s, and Parkinson’s diseases [14]. Increased levels of GSH and CYS have been noted in AIDS-related dementia [15]. The depletion of intracellular aminothiols has also been associated with liver disease, leukemia, diabetes mellitus, and several other disorders [15]. Consequently, the aminothiol levels in physiological systems need to be monitored for further investigation of their specific role as biomarkers [16].

Identification and determination of thiol compounds include methods based on gas chromatography [17] and high-performance liquid chromatography (HPLC) coupled with spectrofluorimetric [5,18,19], spectrophotometric [1,3], mass spectrometric [6], or electrochemical [4,20] detection. Capillary electrophoresis coupled with different detection techniques has also been applied [21,22].

Although various analytical methods are known for the determination of thiol compounds in biological matrices such as urine [3,23], human plasma [1,6,24], human blood [15], rabbit eye tissues [8], and rat brain and rat striatum [10,11], new assays are still required as the measurement of thiols is complicated due to their instability in aqueous solutions and tendency to oxidize into disulfides. Consequently, a robust and specific method is still highly desired in the fields of biological and clinical sciences. HPLC is the mainstream and most widespread technique for the determination of thiols in different matrices because of its high repeatability and sensitivity. Since most thiol compounds have no visible or ultraviolet absorption capacity and have no fluorescence properties, chemical derivatization is widely required and usually affects the improvement of the assays’ sensitivity and selectivity.

The purpose of the presented study was to develop a fast, sensitive, one step HPLC method using on-column derivatization with *o*-phthalaldehyde (OPA) and butylamine (B-NH_2_) and spectrofluorimetric detection that allowed for the simultaneous determination of NAC and GSH in biological matrices such as urine, plasma, and brain tissue samples. To confirm the applicability of this method, a validation process was performed as required by the FDA Guidance for Industry Bioanalytical Method Validation [25]. 

## 2. Results and Discussion

The developed method—dedicated for the simultaneous determination of antioxidants such as NAC and GSH—appears to be suitable for the sensitive and robust analysis of various types of matrices. Although many assays for the determination of NAC and GSH in biological matrices have been published, they are usually dedicated to one or two types of matrices. These assays exploit various fluorescent reagents such as N-(1-pyrenyl)maleimide [5], 5-methyl-(2-(m-iodoacetylaminophenyl)benzoxazole [26], OPA [11], 5-bromo-7-fluorobenzo-2-oxa-1,3-diazole-4-sulfonate [24], and 1,3,5,7-tetramethyl-8-phenyl-(2-maleimide)difluoroboradiaza-s-indacene [19]. The compounds above-mentioned are extensively utilized in procedures dedicated for the control of thiol levels in biological samples and are needed for analyte fluorescence labeling. The previously described methods are based on pre-column or post-column derivatization reactions. Utilizing the compounds above-mentioned is usually time consuming, mostly due to the low reactivity that enforces the application of pre- or post-column derivatization modes. Moreover, in the case of post-column derivatization, additional equipment is also required [11,24]. This approach adds one extra step in the sample preparation procedure, and in the case of post-column derivatization, results in wider peaks. In the method described herein, these drawbacks were circumvented by using an on-column derivatization.

In contrast to the protocols described earlier [5,11,19,24,26], in our approach, the derivatization reaction occurs directly in the chromatographic column during the separation process. Such a solution significantly speeds up and simplifies the analytical procedure. 

### 2.1. Reduction of the Disulfide Bonds

In the case of total thiol determination, to maximize the yield of derivatization, a reduction of the disulfide bonds is required. The reduction reaction releases the thiol groups and makes them accessible for the derivatizing reagent. Here, tris-(2-carboxyethyl)phosphine (TCEP) was used as the reducing agent. TCEP is known to be useful during the reduction of disulfide bonds under mild conditions of pH and temperature [3,21]. In our approach, the disulfide bonds were reduced in 10 min at room temperature at pH 7.8. Since TCEP was added to the sample, it was necessary to investigate how its presence affected the on-column derivatization. According to our prediction, the lack of TCEP dramatically decreased the sizes of the chromatographic signals of both the NAC and GSH (see Figure S1 in Supplementary Information). More importantly, the addition of TCEP to the sample did not disturb the on-column derivatization reaction.

### 2.2. Deproteinization 

The addition of inorganic acids (trichloroacetic acid or perchloric acid, PCA) is essential to precipitate the proteins present in various biological matrices such as plasma or tissue homogenates [3,21]. The need for protein elimination in the presented assay results from the type of chromatographic column utilized for the analyses. In this protocol, an effective protein removal was obtained by the addition of PCA to the sample followed by centrifugation. Then, 5 μL of the obtained supernatant was injected in the chromatographic column.

### 2.3. Derivatization and Chromatographic Separation

**Figure 1:**
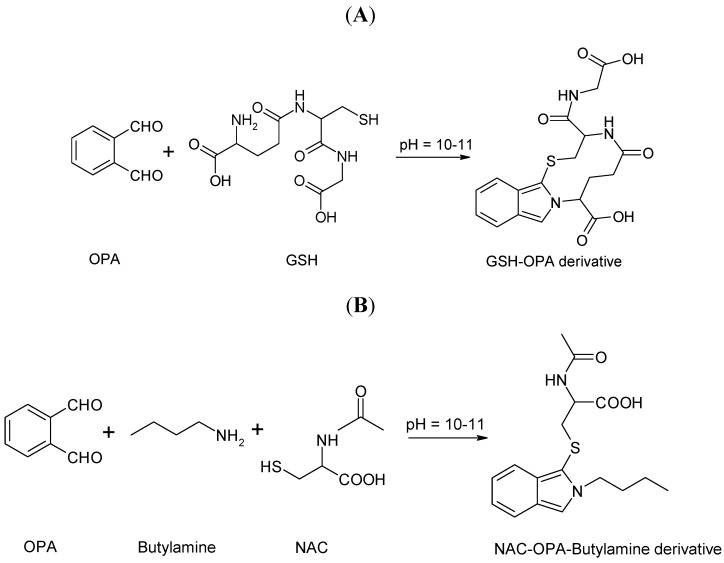
Schemes of the chemical derivatization reactions of GSH with OPA (**A**) and NAC with OPA in the presence of B–NH_2_ (**B**).

The conducted experiments were centered around the development of a sensitive and simple analytical procedure dedicated for the determination of the NAC and GSH in different types of biological matrices. Optimization of the method was carried out with the use of reversed phase HPLC with spectrofluorimetric detection. For signal enhancement and method repeatability, the on-column derivatization was applied. OPA combined with B–NH_2_ were used as the derivatizing reagents. The schemes of the reactions of GSH with OPA and NAC with OPA and B–NH_2_ are shown in Figure 1. On-column derivatization was successfully exploited earlier for HCY and/or methionine determination in plasma, urine, and wine samples [27,28,29] and for the determination of LA in urine samples [30]. The selection of optimal conditions for sample preparation was performed using the urine sample spiked with NAC as well as the plasma and homogenization of brain tissues spiked with GSH and NAC. The amounts of TCEP, OPA, and B–NH_2_ were also optimized.

It is commonly known that the composition of a mobile phase, its pH, and flow rate, play a pivotal role in the separation of the sample components. Under highly alkaline conditions, OPA reacts rapidly with GSH (Figure 1A) and in the presence of B–NH_2_ with NAC (Figure 1B). 

**Figure 2:**
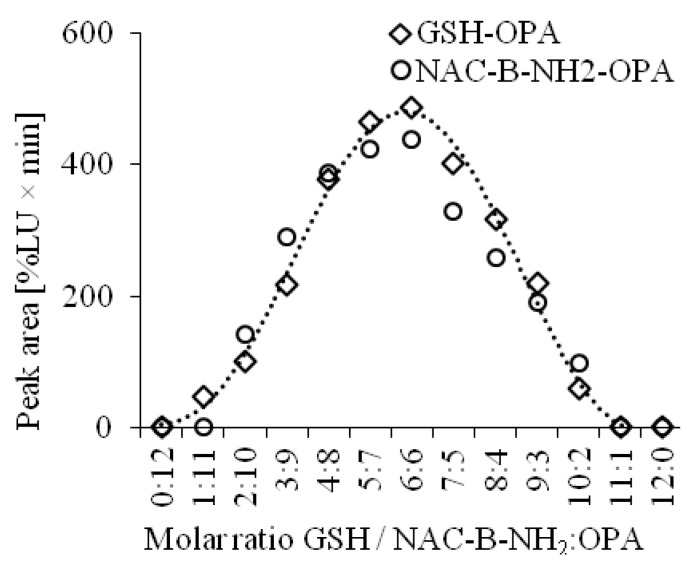
Estimation of the stoichiometric molar ratio by the continuous variation method for the reaction of glutathione with *o*-phthaldialdehyde and reaction of N-acetylcysteine with *o*-phthaldialdehyde in the presence of butylamine.

The chemical structures of the detected analytes have been confirmed in previous studies [31,32]. Additionally, our experiments based on the continuous variation method showed that substrates GSH and OPA react in the molar ratio of 1:1. Similar results were obtained for the reaction of NAC and OPA in the presence of B–NH_2_ (Figure 2). 

**Figure 3:**
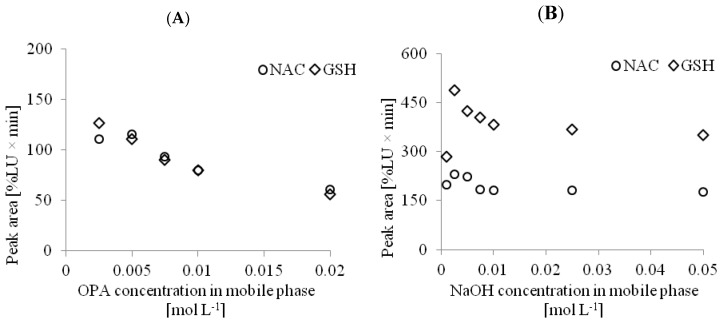

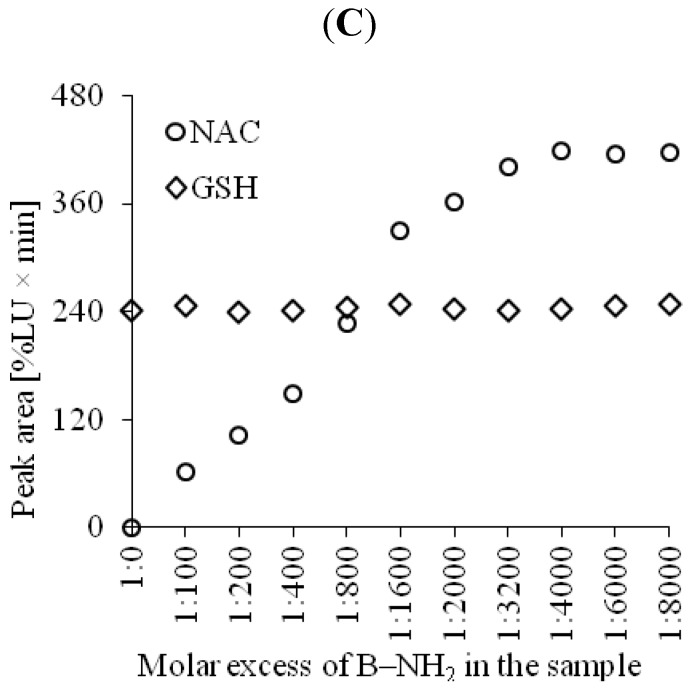
Optimization of the NAC and GSH on-column derivatization reaction performed on the plasma sample. An OPA/NaOH solution was used as the on-column derivatizing agent. (**A**) Optimization of OPA concentration (0.02 mol L^−1^ NaOH, and acetonitrile in the gradient mode described in Section 3.6.2 HPLC Analysis), (**B**) Optimization of NaOH concentration (0.0025 mol L^−1^ OPA and acetonitrile in the gradient mode described in Section 3.6.2 HPLC Analysis), and (**C**) Optimization of the molar excess of B–NH_2_ to NAC and GSH (0.05 mol L^−1^ NaOH, 0.0025 mol L^−1^ OPA, and acetonitrile in the gradient mode described in Section 3.6.2 HPLC Analysis); flow rate 1 mL min^−1^. HPLC analyses were carried out using a reversed phase Hamilton PRP1 column.

Due to the fact that separation and derivatization took place at the same time and under the same conditions in our approach, we focused on these two parameters. The first one was centered around the best conditions for on-column analyte modification. The second concerned the estimation of the best parameters for the separations. It has been shown previously that for on-column derivatization of thiols with OPA, high alkaline conditions are needed [27,28,29,30]. To find the best conditions for the derivatization reaction, we considered the influence of sample deproteinization with the use of PCA on the final conditions of the reaction. According to the protocol described in Section 3.5.1, the preparation of the urine samples did not include the addition of PCA. In this case, the final pH of the solution injected into the column equaled 7.8. For analysis of NAC in the urine samples, the final concentration of NaOH in the mobile phase amounted to 0.025 mol L^−1^. In the case of the determination of NAC and GSH in the plasma or tissue homogenate samples, the concentration of NaOH in the mobile phase was estimated once more due to the sample acidification. It was found that the concentration of NaOH and OPA in this case must be higher to compensate for the acidic environment of the samples after deproteinization by the addition of PCA. For those matrices, 0.05 mol L^−1^ NaOH in the mobile phase was required. Selection of the appropriate conditions for the derivatization reaction for the determination of NAC and GSH in the plasma samples is shown in Figure 3.

**Figure 4:**
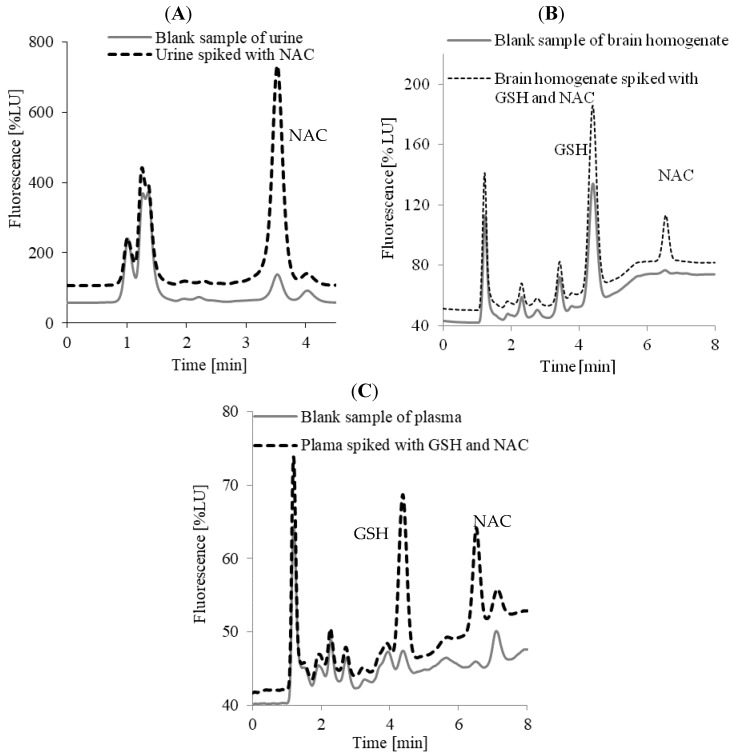
Representative chromatograms for NAC in human urine (**A**), NAC and GSH in pig brain tissue homogenates (**B**), and NAC and GSH in human plasma (**C**) after reduction with TCEP and on-column derivatization with OPA and B–NH_2_. Chromatographic conditions were as described in Section 3.6. HPLC Analysis.

In both cases, under optimized conditions derivatization of GSH and NAC (in the presence of B-NH_2_) occurred immediately, producing the corresponding derivatives. Importantly, simultaneous derivatization and separation significantly shortened the total time of the analytical procedure. The time necessary for the preparation of the urine samples amounted to only 15 min, while for the plasma and brain tissue homogenates it was 25 min. The analysis time was also short and amounted to 4.6 min for the urine samples and 8 min for the plasma and brain tissue homogenates. It turned out that the optimal conditions for NAC determination in the urine samples involved isocratic elution with the mobile phase consisting of acetonitrile and 76% of 0.0025 mol L^−1^ OPA in 0.025 mol L^−1^ NaOH. Under these conditions, the NAC signal was observed at 3.5 min. As can be seen in Figure 4A, the peak NAC was well-spaced from the front of the chromatogram. Moreover, the background of the chromatogram was fairly clean and co-eluting signals from the other matrix components were not observed. Figure 4A shows the chromatograms of the urine and urine spiked with NAC prepared according to the procedure described in Section 3.5.1. For the plasma samples and brain tissue homogenates, a mobile phase containing 0.0025 mol L^−1^ OPA in 0.05 mol L^−1^ NaOH (A) and acetonitrile (B) in gradient elution was used. Under these conditions, the retention times for GSH and NAC were 4.5 min and 6.5 min, respectively. Figure 4B shows the chromatograms of the plasma and plasma spiked with NAC and GSH prepared according to the procedure described in Section 3.5.2. Figure 4C shows the chromatograms of the brain tissue homogenate and brain tissue homogenate spiked with NAC and GSH prepared according to the procedure described in Section 3.5.3. Derivatives of OPA and thiol compounds are known to possess different maximum absorptions. In the presented approach, the optimal excitation and emission wavelengths were 340 nm and 440 nm, respectively (see Figure S2 in Supplementary Information). These detection parameters increase the specificity and sensitivity of the method. At these wavelengths, other analytes containing the thiol group (e.g., HCY, CYS) were not observed (see Figure S3 in Supplementary Information).

### 2.4. Method Validation

#### 2.4.1. LOD and LOQ

Limit of detection (LOD) and limit of quantification (LOQ) were defined as the lowest concentrations giving the signal/noise ratio of 3 and 10, respectively. LOD and LOQ for the developed method concerning the determination of NAC in the urine samples were 0.01 nmol mL^−1^ and 0.02 nmol mL^−1^, respectively. In the case of the brain homogenates and plasma samples, the LOD were 0.02 nmol mL^−1^ and 0.01 nmol mL^−1^ while the LOQ values ranged from 0.05 nmol mL^−1^ and 0.02 nmol mL^−1^ for GSH and NAC, respectively. 

#### 2.4.2. Linearity

For the plasma and urine samples, eight-point calibration plots were constructed for NAC and GSH in triplicate. In both cases, the correlation coefficients were greater than 0.999. For the brain tissue samples, six-point calibration curves were constructed for both analytes in triplicate. Correlation coefficients expressed as R^2^ were greater than 0.998. The calibration data including regression equations are shown in Table 1. 

#### 2.4.3. Accuracy and Precision

To prove the accuracy and precision of the proposed method, known amounts of standard solutions of the analytes were added to plasma, urine and brain tissue homogenate samples, as described in Section 3.8.1. Precision was calculated as the relative standard deviation, whereas accuracy was considered as the percentage of analyte recovery. To estimate the method’s accuracy, the following formula was used:

accuracy (%) = 100% × (measured amount − endogenous content)/added amount.

Measured concentrations were assessed by the application of calibration curves obtained on that occasion. The estimated validation parameters for all analytes and matrices were correct and in line with the requirements dedicated for biological sample analysis [25,33]. Detailed data are presented in Table 2.

#### 2.4.4. NAC and GSH Stability Study

The short-term stability of NAC in urine as well as NAC and GSH in the plasma and brain homogenates at 4 °C and 25 °C was studied chromatographically. Experiments proved that both NAC and GSH in the presence of TCEP were stable for about 80 min at 25 °C independent of the tested matrix. At 4 °C, the stability of both analytes increased up to about 160 min (see Figure S4 in Supplementary Information).

Freeze–thaw studies concerning the stability of the analytes in brain tissue homogenates indicated that the first thawing of the sample resulted in a slight decrease of the NAC and GSH levels. It was also observed that multiple sample thawing strongly affected the analyte content. In the case of GSH, the content of the analyte in the sample decreased over 20% after two cycles of freezing at −20 °C and thawing at 4 °C. These observations firmly indicate that brain tissue homogenates should be analyzed immediately after collection or at most after one thawing (see Figure S5 in Supplementary Information). Similar relationships were observed in the urine and plasma samples.

### 2.5. Application of the Method

**Figure 5:**
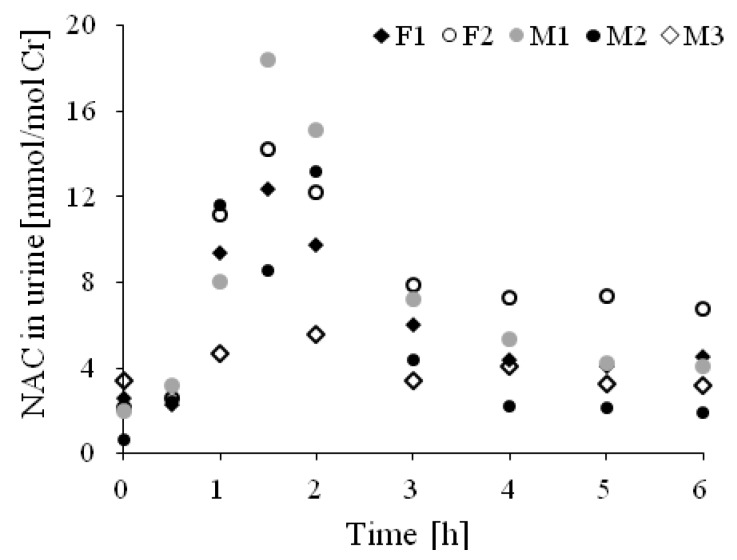
NAC urinary excretion, the NAC concentration in urine samples normalized against Cr, and after the oral administration of 200 mg of NAC in the form of a commercially available pharmaceutical formulation named ACC 200 mg. Chromatographic conditions were as described in Section 3.6. HPLC Analysis.

The methodology described above was applied to the analysis of urine, plasma, and brain tissue samples. Regarding urine samples, the trial concerned the NAC determination and kinetics of its excretion. The changes in the NAC level in urine were measured in the urine samples donated by apparently healthy volunteers treated with a pharmaceutical formulation containing 200 mg of NAC. At the beginning of the test, urine samples were collected every 30 min, later every hour. In order to facilitate a comparison of the results, the NAC contents were normalized against creatinine (Cr). The obtained results allowed us to conclude that the maximum amount of NAC was excreted within 1.5 h after oral administration and gradually dropped up to 3 h (Figure 5). After that, the amount of NAC in urine became constant. The obtained results were similar to those observed by other studies [34].

The proposed method was also adapted to the plasma and brain samples. Five different pig brain tissues were homogenized and analyzed and the plasma and urine samples donated from 13 people were tested for NAC and GSH content. In the urine samples, the average NAC content was 2.7 ± 1.7 mmol mol^−1^ Cr. In the analyzed homogenates of the brain tissues, the average contents of NAC and GSH were 4.2 ± 0.8 nmol mL^−1^ and 14.2 ± 7.3 nmol mL^−1^, respectively. For plasma, the average levels of NAC and GSH were 8.2 ± 1.2 nmol mL^−1^ and 9.6 ± 3.4 nmol mL^−1^, respectively. These data are similar to those published previously [8,24,34]. 

The most important validation data of the newly developed method were compared with the data presented in previously published HPLC approaches based on spectrofluorimetric detection dedicated for the determination of NAC and GSH in biological samples (Table 3). 

The main advantage of the elaborated assay is a shortening of the total time of the analytical protocol and simplification of the sample preparation procedure caused by application of B–NH_2_ as a conjugative reagent to the on-column simultaneous derivatization and separation of the analytes. This method does not require additional procedures such as overheating or extraction, and avoids random errors. The low cost of the overall analyses, the small amounts of derivatizing reagents used for the analytical procedure, the easiness of the protocol, and the versatility in terms of the type of matrix are additional benefits of the presented assay. Considering the antioxidant role of GSH and NAC in mammal bodies [11,12,13], the presented method seems to be very useful for the determination of these analytes in biological samples such as urine, plasma, and tissues. To prove the benefits of the presented protocol, a comparison of the analytical protocols based on OPA derivatization for the determination of NAC and GSH in biological and pharmaceutical samples is presented in Table 4.

## 3. Materials and Methods

### 3.1. Chemicals and Reagents

*N*-acetylcysteine, oxidized glutathione, *o*-phthaldialdehyde, butylamine, creatinine, and tris-(2-carboxyethyl)phosphine were received from the Sigma Aldrich Company (St. Louis, MO, USA). The HPLC gradient grade acetonitrile used for chromatographic analysis, hydrochloric acid (HCl) utilized for the standard solution preparation, sodium hydrogen phosphate heptahydrate (Na_2_HPO_4_·7H_2_O) and sodium dihydrogen phosphate dihydrate (NaH_2_PO_4_·2H_2_O) required for the phosphate buffer preparation, and sodium hydroxide (NaOH) applied for the mobile phase preparation were all purchased from J.T. Baker (Deventer, The Netherlands). Perchloric acid (PCA) was from Merck (Darmstadt, Germany). Deionized water was produced in our laboratory. 

### 3.2. Instrumentation

All analyses were performed on a 1200 Series HPLC system (Agilent Technologies, Waldbronn, Germany) equipped with a quaternary pump, vacuum degasser, autosampler, module of temperature control, and spectrofluorometric detector. All analyses were controlled by HP ChemStation software. A Hamilton PRP-1 column from Energy Way, Reno, NV, USA, parameters: 150 × 4.6 mm, 5 μm was used for the analyte separation. Water used for the mobile phase preparation was distilled with the use of a Milli-QRG system from Millipore in Vienna, Austria. The pH of the phosphate buffer and mobile phases was controlled using a HI 221 pH meter, model Hanna Instruments, Woonsocket, RI, USA. Precipitated proteins were separated using a Hettich Micro 200R centrifuge (Hettich Zentrifugen, Tuttlingen, Germany). For sample homogenization, an IKA T10 basic homogenizer (IKA^®^-Werke GmbH&Co. KG, Staufen, Germany) was used.

### 3.3. Biological Matrices

#### 3.3.1. Urine

The urine samples were collected from healthy donors from different ages and sexes (three males and two females, 25–45 years old). Samples were collected in two sets: as the first morning urine from subjects non supplemented with NAC before sample collection, and as samples from volunteers supplemented with 200 mg of NAC in the form of a commercially available pharmaceutical formulation named ACC 200 mg. After supplementation, samples were collected at five time intervals: 0.0, 0.5, 1, 1.5, 2, 2.5, 4.0, and 6.0 h after supplementation. The ACC 200 mg was purchased from the local pharmacy. Urine samples were used for the analyses without delay or were stored at −80 °C, if needed.

#### 3.3.2. Brain Tissues

Pig brain tissues were purchased from the local market. The brain was divided into portions and stored in a freezer at −80 °C.

#### 3.3.3. Human Plasma

We studied the plasma samples donated by 13 volunteers. Blood was collected into vacutainer tubes containing EDTA by venipuncture, immediately placed on the ice, and centrifuged at 800× *g* for 15 min at room temperature. Plasma was used for the analyses without delay or stored at −80 °C. 

All investigations were performed after approval by the Ethical Committee of the University of Łódź, the decision identification code: 9/KBBN-UŁ/II/2017 approved on November 6, 2017. Informed consent forms were obtained from all volunteers involved into the project.

### 3.4. Stock Solutions

Stock solutions of 0.1 mol L^−1^ NAC and 0.05 mol L^−1^ oxidized GSH were prepared in 0.1 M HCl and kept at 4 °C for several days without noticeable change of the analyte content. The working solutions were prepared by dilution with water as needed. Stock solutions of 0.2 and 1.0 mol L^−1^ B–NH_2_ in water were prepared fresh every day. Fresh stock solutions of TCEP (0.25 mol L^−1^) were prepared daily in 0.5 mol L^−1^ Tris/HCl buffer pH 9 (urine samples) or 0.2 mol L^−1^ phosphate buffer pH 7.8 (plasma and brain tissue samples). 

### 3.5. Procedure for Biological Samples Preparation 

#### 3.5.1. Urine

As the donated urine samples did not contain proteins, the samples were prepared directly in HPLC vials. Urine (50 µL) was diluted with 200 µL of Tris/HCl buffer (pH 9, 0.5 mol L^−1^), treated with 5 µL of TCEP (0.25 mol L^−1^ in Tris/HCl buffer) and kept for 10 min at room temperature. Next, 60 µL of 1 mol L^−1^ B–NH_2_ was added. Then, the samples were diluted with water to a final volume 500 µL. A total of 5 μL of the final analytical solution was injected into the HPLC column. To estimate the Cr in urine samples, a previously published method was exploited [23].

#### 3.5.2. Brain Tissue 

The first step in preparing the brain tissue samples was sample homogenization. For 1 g of tissue, 10 mL of cold phosphate buffer 0.2 mol L^−1^ pH 7.8 was added and homogenized for 30 s on ice and cooled at 4 °C. Next, 100 µL of the homogenate was transferred to a tube, and for the reduction of the disulfide bonds, 3 µL of TCEP solution (0.25 mol L^−1^ in phosphate buffer, pH 7.8, 0.2 mol L^−1^) was added. After 10 min of reduction, 80 µL of 0.2 mol L^−1^ B–NH_2_, as a derivatization conjugative agent and 15 µL of 3 mol L^−1^ PCA for protein precipitation, were added to the mixture. The mixture was diluted with deionized water to the final volume of 400 µL. The precipitated proteins were removed by centrifugation (15,000× *g*, 10 min, 10 °C) and the supernatant (5 μL) was injected into the chromatographic column.

#### 3.5.3. Human Plasma

**Figure 6:**
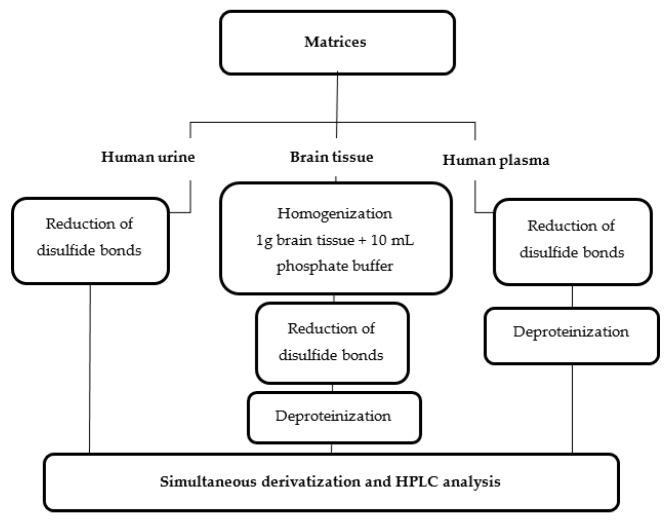
Diagram of the sample preparation for the determination of NAC in urine as well as NAC and GSH in the plasma and brain tissues samples.

A total of 50 µL of plasma was diluted with 100 µL of 0.2 mol L^−1^ pH 7.8 phosphate buffer and treated with 3 μL of 0.25 mol L^−1^ TCEP in phosphate buffer, pH 7.8, 0.2 mol L^−1^ for 10 min. In the next step, 80 µL of 0.2 mol L^−1^ B–NH_2_ was added. To precipitate the plasma proteins, 3 mol L^−1^ PCA (15 µL) was added into the mixture. Next, an appropriate amount of deionized water was added to obtain the final volume of 400 µL. The precipitated proteins were removed by centrifugation (15,000× *g*, 10 min, 10 °C). As previously stated, 5 μL of the final sample was injected into the chromatographic column. The scheme of the preparation of brain tissues, urine, and plasma samples is presented below (Figure 6).

### 3.6. HPLC Conditions for Determination of NAC and GSH 

#### 3.6.1. Urine

Due to the highly alkaline properties of the proposed mobile phase, a reversed phase Hamilton PRP-1 column was used for the chromatographic separation of NAC in urine. The analyte was eluted by the mobile phase containing 76% 0.0025 mol L^−1^ OPA in 0.025 mol L^−1^ NaOH as a component and 24% acetonitrile using isocratic elution. The flow rate of the mobile phase was 1 mL min^−1^. For the detection of NAC, wavelengths of 340 nm for the excitation and 440 nm the emission were used. Separation of the analyte was performed at room temperature. The analysis was five minutes long.

#### 3.6.2. Brain Tissues and Human Plasma

The chromatographic separation of NAC and GSH was obtained in 10 min. The analytes were eluted by the mobile phase containing 0.0025 mol L^−1^ OPA in 0.05 mol L^−1^ NaOH (A) and acetonitrile (B) with the gradient elution as follows: 0–3 min, 22–30% (B); 3–6 min, 30% (B), 6–8 min, 30–22% (B). For column equilibration, a 2 min post time was used. Flow rate of the mobile phase was 1 mL min^−1^. For detection of the NAC and GSH, wavelengths for excitation at 340 nm and emission at 440 nm were used. Separations were performed at room temperature. 

### 3.7. Calibration

The calibration standard solutions were prepared in triplicate. The peak area was used for quantification of the analytes.

#### 3.7.1. Urine

Calibration curves in the validation process were prepared as follows: 10 µL of the working standard solutions of NAC were added to the 50 µL urine sample to provide the final concentration of 5, 10, 20, 50, 100, 150, and 200 nmol mL^−1^ urine and mixed with 190 µL of Tris/HCl buffer (pH 9, 0.5 mol L^−1^). Then, the samples were processed according to the procedure described in Section 3.5.1.

#### 3.7.2. Brain Tissue

Brain tissue homogenate samples (100 µL) were spiked with increasing amounts of working solutions of GSH and NAC to provide the final concentrations for GSH of 0.5, 2.5, 5, 10, and 15 nmol mL^−1^ homogenates and for NAC 0.5, 2, 3, 4, and 5 nmol mL^−1^ homogenates. Then, the samples were processed according to the procedure described in Section 3.5.2.

#### 3.7.3. Human Plasma

For the preparation of the calibration standards for NAC and GSH determination in plasma, portions of 50 µL of the samples were spiked with 10 µL of increasing amounts of working standard solutions to provide the final concentrations of GSH 0.5, 1.0, 5.0, 7.5, 10.0, and 15.0 nmol mL^−1^ plasma and of NAC 0.25, 0.5, 1.0, 3.0, 4.0, and 5.0 nmol mL^−1^ plasma. Then, the samples were processed according to the procedure described in Section 3.5.3. 

### 3.8. Validation Process and Stability Study

#### 3.8.1. Validation Process

The proposed method was validated according to the guidelines for biological sample analysis [25]. The protocol of the validation procedure involved the LOD, LOQ, linearity range, and stability studies as well as accuracy and precision investigations. Precision and accuracy were determined by the addition of known amounts of analyte standards to the samples. Three concentrations representing the entire range of the calibration curves were studied: 5.0, 50.0 and 200.0 mL^−1^ urine for NAC; 0.5, 2.0 and 5.0 mL^−1^ brain homogenate for NAC and 0.5, 5.0 and 15.0 mL^−1^ brain homogenate for GSH, 0.25, 1.0 and 5.0 mL^−1^ plasma for NAC and 0.5, 5.0 and 15.0 mL^−1^ plasma for GSH. Peaks of the analytes were identified through a comparison of their retention times with authentic standards. Stability of the chromatographic conditions was characterized by reproducibility of the retention times.

During the process of validating the new method, the linear range in different matrices for both analytes was studied. Additionally, the lowest limit of detection and the upper limit of detection were indicated. In the case of a higher concentration of these analytes, an additional dilution of the sample is required, which prevents chromatographic column overloading.

#### 3.8.2. Stability Study

Urine, brain tissue homogenate, and plasma samples were prepared in the same way as described in Section 3.5. Samples were stored at 4 °C or at 25 °C. Every 5 μL of the final mixtures were injected in the HPLC column and analyzed under the chromatographic conditions described in Section 3.6. 

Freeze–thaw studies were also performed using the brain tissue homogenates. The brain homogenate prepared according to the procedure described in Section 3.5.2 was thawed and frozen four times, and at each time, aliquots of the final analytical solution were assayed for NAC and GSH content. 

## 4. Conclusions

In this paper, we proposed a new method for the simultaneous separation and determination of NAC and GSH in different biological matrices, namely human urine, human plasma as well as pig brain tissues. The assay is based on the on-column derivatization mode of chemical modification elaborated in our laboratory. The presented methodology exhibits some advantages when compared to other previously published reversed phase HPLC based methods. Our approach significantly simplifies and reduces the time taken by the sample preparation step. In the case of urine specimens, only dilution of the sample and the reduction of disulfide bonds are needed. The method exploits different chemistries of NAC and GSH during reaction with OPA, resulting in different chromatographic properties of their derivatives. From an analytical point of view, our test is simple, fairly fast, sensitive, and does not require large sample volumes. The validation parameters including linearity, precision, and accuracy were within the rules for biological samples. Furthermore, the proposed method can be applied for the analysis of different biological matrices for NAC and GSH in every laboratory. To the best of our knowledge, the on-column derivatization utilizing B–NH_2_ as a conjugative reagent for the determination of NAC and GSH in biological matrices has not been proposed as yet. 

## Figures and Tables

**Table 1:** Validation data.

Matrix	Linear Range [nmol mL^−1^ Sample]	Regression Equation	R^2^	RSD [%]		Recovery [%]	
				Min.	Max.	Min.	Max.
Urine NAC	5.0–200.0	y = 41.6x + 1103.9	0.999	1.1	11.5	93.7	112.0
Brain tissue NAC GSH	0.5–5.0 0.5–15	y = 55.4x + 44.1 y = 40.2x + 1216.9	0.998 0.998	3.1 0.5	6.9 5.0	92.1 98.0	103.0 106.7
Plasma NAC GSH	0.25–5.0 0.5–15.0	y = 37.5x + 10.9 y = 20.5x + 46.0	0.999 0.999	1.2 0.5	9.0 9.4	94.0 98.7	106.0 105.4

**Table 2:** Accuracy and precision.

Matrix	Analyte	Concentration [nmol mL^−1^ Sample]	Precision [%]		Accuracy [%]	
			Intra-Day	Inter-Day	Intra-Day	Inter-Day
Urine	NAC	5	8.4	5.4	100.9	113.4
		50	0.8	0.8	99.1	98.9
		200	0.3	2.2	95.3	93.8
Brain tissue	NAC	0.5	14.8	2.1	105.7	114.9
		2	4.6	10.6	94.9	91.3
		5	5.3	1.9	95.3	98.3
	GSH	0.5	4.8	2.4	93.9	90.9
		5	2.8	2.8	102.4	113.7
		15	5.2	1.8	115.5	116.2
Plasma	NAC	0.25	10.9	8.7	110.8	114.9
		1	4.2	1.2	97.8	91.7
		5	4.8	11.5	99.1	106.0
	GSH	0.5	5.0	9.7	91.6	100.1
		5	1.1	3.5	98.7	99.7
		15	1.9	6.1	99.9	105.8

**Table 3:** Comparison of the analytical protocols based on HPLC methods coupled with spectrofluorimetric detection for the determination of NAC in urine samples and for the determination of NAC and GSH in plasma and brain homogenate samples.

Parameters	HPLC-FLD Ref. [5]	HPLC-FLD Ref. [11]	HPLC-FLD Ref. [19]	HPLC-FLD Ref. [24]	HPLC-FLD Ref. [26]	HPLC-FLD Proposed Methods		
Matrices type	Plasma Tissue	Rat striatum	Pig liver	Plasma	Urine	Urine	Plasma	Brain tissues
Derivatization type	Pre- column	Pre- column	Pre- column	Pre- column	Pre- column	On-column		
Derivatization reagent	NPM	OPA	TMPAB-o-M	SBD-BF	MIPBO	OPA		
Derivatization time [min]	5	5	6	30	35	Immediately, during analysis		
LOD [nmol L^−1^] NAC GSH	5 No data	No data 2.7	No data No data	No data 20	No data 0.35	10 20		
LOQ [nmol L^−1^] NAC GSH	25 No data	No data 8.2	No data No data	No data 50	No data No data	20 50		
Linearity [µmol L^−1^] NAC GSH	0.025–5.0 No data	No data 0.05–0.50	0.001–0.5 No data	No data 0.05–20.0	No data No data	5.0–200.0 No data	0.25–5.0 0.5–15.0	0.5–5.0 0.5–15.0
Analysis time [min]	20	10	6	40	16	5	8	

NPM = N-(1-pyrenyl)maleimide; TMPAB-o-M = 1,3,5,7-tetramethyl-8-phenyl-(2-maleimide) difluoroboradiaza-s-indacene; SBD-BF = ammonium 5-bromo-7-fluorobenzo-2-oxa-1,3-diazole-4-sulfonate; MIPBO = 5-methyl-(2-(m-iodoacetylaminophenyl)benzoxazole.

**Table 4:** Comparison of the analytical protocols based on OPA derivatization for the determination of NAC or/and GSH in biological or pharmaceutical samples.

Parameters	Ref. [35]	Ref. [36]	Ref. [37]	Ref. [38]	Our Method
Method principles	Pre-column derivatization; HPLC-FLD	Pre-column derivatization; HPLC-FLD	Pre-column derivatization; HPLC-FLD	Post-column derivatization; HILIC-FLD	On-column derivatization; HPLC-FLD
Method application	Plasma/Blood	Plasma/Blood	Pharmaceutical formulations	Pharmaceutical formulations	Plasma/Urine/Tissue homogenates
Sample volume [µL]	100	200	-	-	P: 50 U: 50 T: 100
Detected analytes	GSH, CYS, HCY	GSH	NAC	NAC	GSH, NAC
Detection [nm] λex λem	360 470	350 420	335 420	345 450	340 440
LOD [nmol L^−1^] For NAC For GSH	no data 3.3	no data 1	5000 no data	no data no data	10 20
Linearity					Plasma:
For NAC For GSH	no data 10–100	no data 1–20	10–40 no data	12.3–91.9 no data	0.25–5.0 0.5–15.0 Urine: 5.0–200.0 No data Tissue: 0.5–5.0 0.5–15.0
Derivatization reagent	OPA/2-ME	OPA	OPA/isoleucine	OPA	OPA/B–NH_2_
Total time of the protocol [min]	60 min + 20 min for column equilibration	55 min	50 min	45 min	P: 33 min U: 21 min T: 33 min

2-ME = 2-mercaptoethanol.

## Supplementary Materials

Supplementary materials can be found at https://www.mdpi.com/1422-0067/20/13/3340/s1.

## Abbreviations

B-NH_2_	Butylamine
Cr	Creatinine
CYS	Cysteine
GSH	Glutathione
HCY	Homocysteine
HPLC	High-performance liquid chromatography
NAC	*N*-acetylcysteine
OPA	*o*-phthaldialdehyde
PCA	Perchloric acid
ROS	Reactive oxygen species
TCEP	tris-(2-carboxyethyl)phosphine

## References

[1] Głowacki R., Bald E. (2009). Determination of *N*-acetylcysteine and main endogenous thiols in human plasma by HPLC with ultraviolet detection in the form of their *S*-quinolinium derivatives. J. Liq. Chrom. Rel. Technol..

[2] Özyürek M., Baki S., Güngör N., Celik S.E., Güclü K., Apak R. (2012). Determination of biothiols by a novel on-line HPLC-DTNB assay with post-column detection. Anal. Chim. Acta.

[3] Kuśmierek K., Chwatko G., Głowacki R., Bald E. (2009). Determination of endogenous thiols and thiol drugs in urine by HPLC with ultraviolet detection. J. Chrom. B.

[4] Hannan P.A., Khan J.A., Iqbal Z., Ullah I., Rehman W.U., Rehman M., Nasir F., Khan A., Khan I., Muhammad S. (2015). Simultaneous determination of endogenous antioxidants and malonodialdehyde by RP-HPLC coupled with electrochemical detector in serum samples. J. Liq. Chrom. Rel. Technol..

[5] Wu W., Goldstein G., Adams C., Matthews R.H., Ercal N. (2006). Separation and quantification of *N*-acetyl-L-cysteine and *N*-acetyl-cysteine-amide by HPLC with fluorescence detection. Biomed. Chromatogr..

[6] Celma C., Allue J.A., Prunonosa J., Peraire C., Obach R. (2000). Determination of *N*-acetylcysteine in human plasma by liquid chromatography coupled to tandem mass spectrometry. J. Chrom. A.

[7] Samuni Y., Goldstein S., Dean O.M., Berk M. (2013). The chemistry and biological activities of *N*-acetylcysteine. Biochim. Biophys. Acta.

[8] Nozal M.J., Bernal J.L., Toribio L., Marinero P., Moral O., Manzanas L., Rodriguez E. (1997). Determination of glutathione, cysteine and *N*-acetylcysteine in rabbit eye tissues using high-performance liquid chromatography and post-column derivatization with 5, 5’-dithiobis(2-nitrobenzoic acid). J. Chromatogr. A.

[9] Bailey B., Waraska J., Acworth I. (2009). Direct determination of tissue aminothiol, disulfide and thioether levels using HPLC-ECD with a novel stable boron-doped diamond working electrode, advanced protocols in oxidative stress II. Methods Mol. Biol..

[10] Xu F., Wang L., Gao M., Jin L., Jin J. (2002). Amperometric determination of glutathione and cysteine on a Pd-IrO_2_ modified electrode with high performance liquid chromatography in rat brain microdialysate. Anal. Bioanal. Chem..

[11] Gawlik M., Krzyżanowska W., Gawlik M.B., Filip M. (2014). Optimization of determination of reduced and oxidized glutathione in rat striatum by HPLC method with fluorescence detection and pre-column derivatization. Acta Chromatogr..

[12] Dean O., Giorlando F., Berk M. (2011). *N*-acetylcysteine in psychiatry: Current therapeutic evidence and potential mechanisms of action. J. Psychiatry Neurosci..

[13] Shahripour R.B., Harrigan M.R., Alexandrov A.V. (2014). *N*-acetylcysteine (NAC) in neurological disorders: Mechanisms of action and therapeutic opportunities. Brain Behav..

[14] Wu G., Fang Y.Z., Yang S., Lupton J.R. (2004). Glutathione metabolism and its implications for health. J. Nutr..

[15] Ivanov A.R., Nazimov I.V., Baratova L.A. (2000). Qualitative and quantitative determination of biologically active low-molecular-mass thiols in human blood by reversed-phase high-performance liquid chromatography with photometry and fluorescence detection. J. Chromatogr. A.

[16] Elgawish M.S., Kishikawab N., Kuroda N. (2015). Quinones as novel chemiluminescent probes for the sensitive and selective determination of biothiols in biological fluids. Analyst.

[17] Capitan P., Malmezat T., Breuille D., Obled C. (1999). Gas chromatographic-mass spectrometric analysis of stable isotopes of cysteine and glutathione in biological samples. J. Chromatogr. B: Biomed. Sci. Appl..

[18] Liang S., Wang H., Zhang Z., Zhou Y.Y., Zhang H.S. (2001). Spectrofluorimetric determination of thiols by use of N-[P-(2-benzoxazolyl)-phenyl]maleimide. Fresenius J. Anal. Chem..

[19] Guo X.F., Zhao P.X., Wang H., Zhang H.S. (2011). Simple and rapid determination of thiol compounds by HPLC and fluorescence detection with 1, 3, 5, 7-tetramethyl-8-phenyl-(2-maleimide)difluoroboradiaza-s-indacene. J. Chromatogr. B Analyt. Technol. Biomed. Life Sci..

[20] Khan M.I., Iqbal Z. (2011). Simultaneous determination of ascorbic acid, aminothiols, and methionine in biological matrices using ion-pairing RP-HPLC coupled with electrochemical detector. J. Chromatogr. B Analyt. Technol. Biomed. Life Sci..

[21] Kuśmierek K., Chwatko G., Głowacki R., Kubalczyk P., Bald E. (2011). Ultraviolet derivatization of low-molecular-mass thiols for high performance liquid chromatography and capillary electrophoresis analysis. J. Chromatogr. B Analyt. Technol. Biomed. Life Sci..

[22] Duan Y.J., Zhang L.Y., Guo X.F., Wang H. (2016). A CE-LIF method based on long wavelength fluorescence labeling for the analysis of thiols in human urine. Electrophoresis.

[23] Kuśmierek K., Głowacki R., Bald E. (2006). Analysis of urine for cysteine, cysteinylglycine, and homocysteine by high-performance liquid chromatography. Anal. Bioanal. Chem..

[24] Cevasco G., Piątek A.M., Scapolla C., Thea S. (2010). An improved method for simultaneous analysis of aminothiols in human plasma by high-performance liquid chromatography with fluorescence detection. J. Chromatogr. A.

[25] FDA Guidance for Industry Bioanalytical Method Validation. http://www.fda.gov/downloads/Drugs/Guidance/ucm070107.pdf.

[26] Liang S.C., Wang H., Zhang Z.M., Zhang H.S. (2005). Determination of thiol by high-performance liquid chromatography and fluorescence detection with 5-methyl-(2-(m-iodoacetylaminophenyl)benzoxazole. Anal. Bioanal. Chem..

[27] Borowczyk K., Chwatko G., Kubalczyk P., Jakubowski H., Kubalska J., Głowacki R. (2016). Simultaneous determination of methionine and homocysteine by on-column derivatization with *o*-phthaldialdehyde. Talanta.

[28] Głowacki R., Borowczyk K., Bald E., Jakubowski H. (2010). On-column derivatization with *o*-phthaldialdehyde for fast determination of homocysteine in human urine. Anal. Bioanal. Chem..

[29] Głowacki R., Borowczyk K., Bald E. (2012). Fast analysis of wine for total homocysteine content by high-performance liquid chromatography. Amino Acids.

[30] Borowczyk K., Olejarz P., Chwatko G. (2019). Application of simultaneous separation and derivatization for the determination of α-lipoic acid in urine samples by high performance liquid chromatography with spectrofluorimetric detection. Biomed. Chromatogr..

[31] Tsikas D., Sandmann J., Holzberg D., Pantazis P., Raida M., Fro¨lich J.C. (1999). Determination of *S*-nitrosoglutathione in human and rat plasma by high-performance liquid chromatography with fluorescence and ultraviolet absorbance detection after pre-column derivatization with *o*-phthalaldehyde. Anal. Biochem..

[32] Neuschwander-Tetri B.A., Roll F.J. (1989). Glutathione measurement by high-performance liquid chromatography separation and fluorometric detection of the glutathione-*ortho*phthalaldehyde adduct. Anal. Biochem..

[33] European Medicines Agency (EMA), Committee for Medicinal Products for Human Use (CHMP) Guideline on Bioanalytical Method Validation. https://www.ema.europa.eu/en/documents/scientific-guideline/guideline-bioanalytical-method-validation_en.pdf.

[34] Kuśmierek K., Bald E. (2008). Determination of *N*-acetylcysteine and thioglycolic acid in human urine. Chromatographia.

[35] Pozdeev V.K., Pozdeyev N.V. (2010). Determination of total aminothiols and neuroactive amino acids in plasma by high performance liquid chromatography with fluorescence detection. Biochem. (Mosc.) Suppl. Ser. B Biomed. Chem..

[36] Kanďár R., Vrbová M., Čandová J. (2013). An assay of total glutathione and glutathione disulfide in human whole blood and plasma using a high performance liquid chromatography with fluorescence detection. J. Liq. Chromatogr. Relat. Technol..

[37] Concha-Herrera V., Torres-Lapasió J.R., García-Alvarez-Coque M.C. (2004). Chromatographic determination of thiols after pre-column derivatization with *o*-phthalaldehyde and isoleucine. J. Liq. Chromatogr. Relat. Technol..

[38] Douša M. (2018). The determination of pharmaceutically active thiols using hydrophilic interaction chromatography followed post-column derivatization with *o*-phthaldialdehyde and fluorescence detection. J. Pharm. Biomed. Anal..

