# Supplementary material for: Application of Butylamine as a Conjugative Reagent to On-Column Derivatization for the Determination of Antioxidant Amino Acids in Brain Tissue, Plasma, and Urine Samples
Source: Int J Mol Sci. 2019 Jul 7;20(13):3340. doi: 10.3390/ijms20133340 (PMC6651812; doi:10.3390/ijms20133340)
Supplement: Supplementary file 1 [file ijms-20-03340-s001.pdf]

Supplementary Information

Application of butylamine as a conjugative reagent to on-column derivatization for the determination of antioxidant amino acids in brain tissue, plasma and urine samples

Kamila Borowczyk*, Patrycja Olejarz, Adrianna Kamińska, Rafał Głowacki,

Grażyna Chwatko

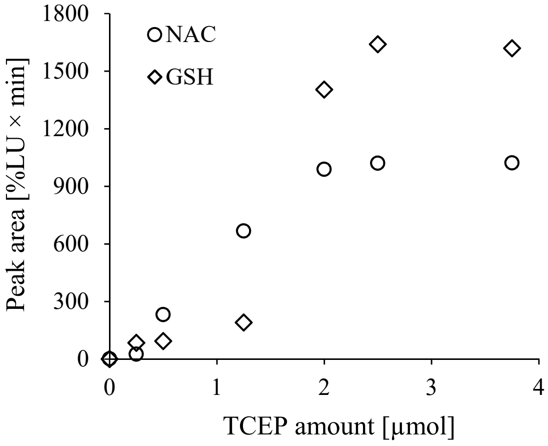

Figure S1. The influence of TCEP excess on NAC and GSH signals after derivatization with OPA at the presence of B-NH_2.

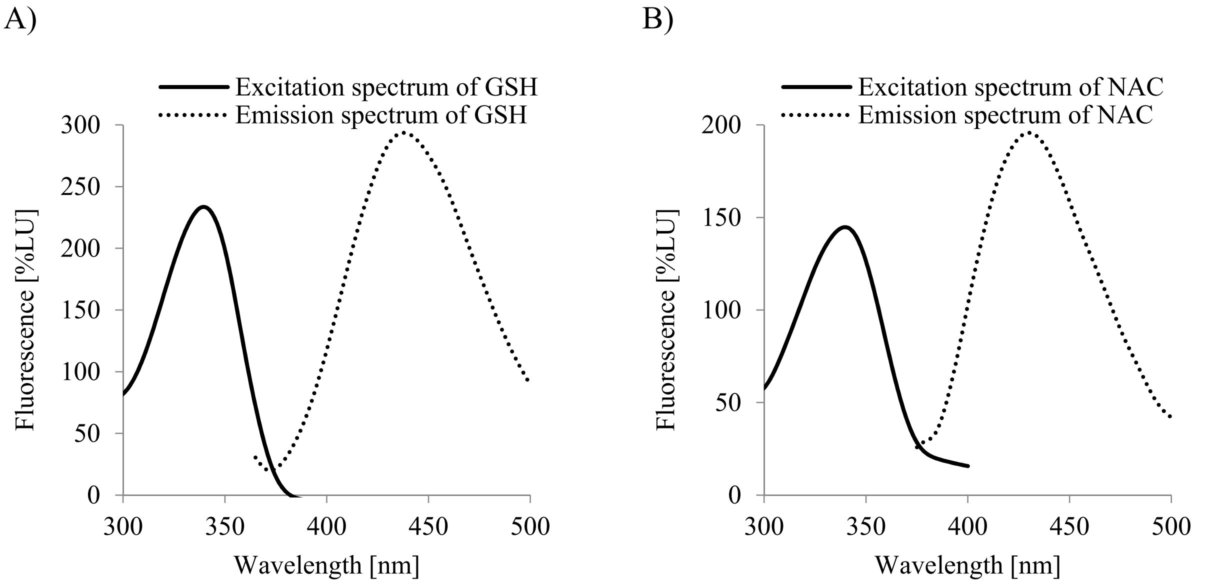

Figure S2. Fluorescence excitation and emission spectra of the GSH-(B-NH_2)-OPA derivative (A) and NAC-(B-NH_2)-OPA derivative (B) after on-column derivatization.

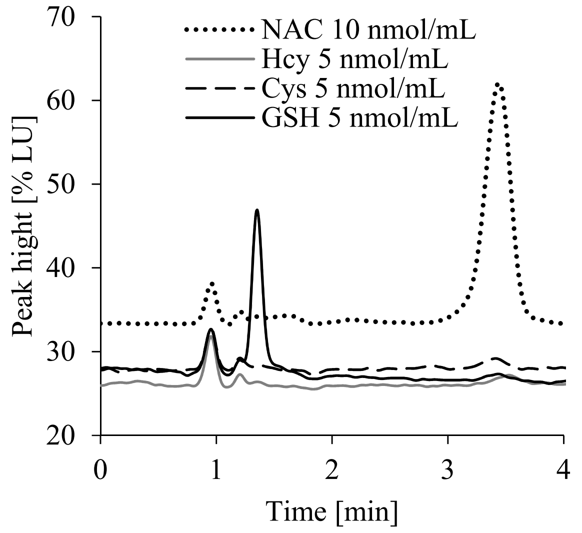

Figure S3. Chromatograms of standard solutions of endogenous aminothiols: N-acetylcyteine (dotted line), homocysteine (solid gray line), cysteine (dashed line), oxidized glutathione (solid black line) after reduction with TCEP and on-column derivatization with OPA and B-NH_2. Chromatographic conditions: mobile phase 76% of 0.0025 mol L^{-1} OPA in 0.025 mol L^{-1} NaOH and 24% of acetonitrile, isocratic elution. The flow rate of the mobile phase was 1 mL min^{-1}. For the detection 340 nm as excitation and 440 nm as emission wavelengths were used. Chromatographic analysis was performed at room temperature.

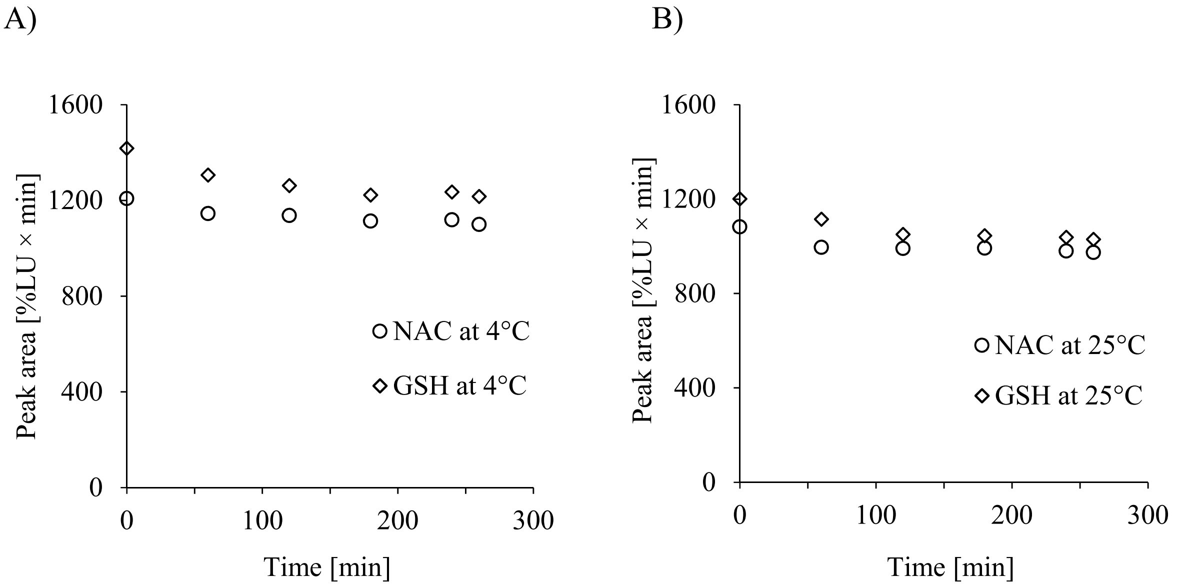

Figure S4. Stability of NAC and GSH at the presence of TCEP and $B\text{-}NH_2$ in a brain tissue homogenate sample stored at 4°C (A) and at 25°C (B).

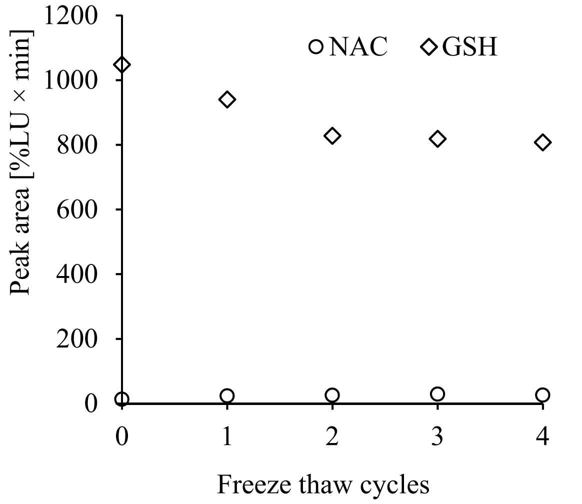

Figure S5. Stability of NAC and GSH in a brain tissue homogenate sample after four cycles of freezing at -20°C and thawing at 4°C.
